# Comprehensive Pan-cancer Analysis and Experimental Verification of EGLN Family: Potential Biomarkers in Cervical Cancer

**DOI:** 10.2174/0115680096362252250527060004

**Published:** 2025-06-03

**Authors:** Dongli Zhang, Ruifang Fu, Guixia Sun, Junfang Yan, Xiaofeng Yang

**Affiliations:** 1 Department of Obstetrics and Gynecology, The First Affiliated Hospital of Xi’an Jiaotong University, Xi’an, Shanxi 710061, P.R. China;; 2 Department of Obstetrics and Gynecology, Huaihe Hospital of Henan University, Henan, Kaifeng 475000, P.R. China

**Keywords:** Hypoxia, pan-cancer, EGLN family, bioinformatics analysis, biomarker discovery

## Abstract

**Background:**

Hypoxia plays a crucial role in malignant tumor formation, primarily mediated by hypoxia-inducible factors (HIFs). Despite extensive research, the complexities and prognostic implications of the EGLN gene family (EGLN1, EGLN2, EGLN3) in cancers remain unclear.

**Methods:**

Utilizing public databases (TCGA, GTEx, TARGET, GEO) and bioinformatics tools, a comprehensive analysis of EGLN genes across various cancer types was conducted. Gene expression, mutation data, stemness scores, and clinical information were integrated to evaluate the mutation landscape, expression levels, and prognostic values of EGLNs. Enrichment and pathway analyses explored EGLN-associated biological processes and functional networks. ssGSEA constructed EGLN scores for prognostic evaluation. Colocalization analysis combined eQTL and GWAS data to investigate genetic variations in cervical cancer. Immunohistochemistry validated EGLN expression in cervical cancer tissues.

**Results:**

EGLN genes showed differential expression across cancer types. EGLN1 overexpression was associated with worse survival in cervical squamous cell carcinoma (CESC), pancreatic adenocarcinoma (PAAD), and neuroblastoma (NB), while EGLN3 was linked to poor survival in CESC, lung adenocarcinoma (LUAD), and kidney cancers. EGLNs also demonstrated varied roles in modulating tumor immune activity and heterogeneity.

**Conclusion:**

This study provides new insights into EGLN biology and identifies EGLN1 as a potential biomarker for cervical cancer.

## 1 INTRODUCTION

Cervical cancer is a heavy disease burden among women globally, with varying incidence rates across regions [1, 2]. The incidence of cervical cancer varies across regions, with higher rates in developing countries due to limited access to screening and vaccination programs [3]. Based on the Global Cancer Statistics 2020, cervical cancer ranks as the fourth most prevalent cancer affecting women worldwide [4]. Cervical cancer treatment typically involves surgical removal, radiation therapy, chemotherapy, emerging targeted therapy, and immunotherapy based on the cancer stage [5]. However, cervical cancer management continues to present significant challenges, including the restricted availability of screening and vaccination initiatives, cultural obstacles, and disparities in healthcare access [6, 7]. Timely detection and intervention with Pap tests are crucial to achieving favorable prognoses based on clinical guidelines [8-10]. Nevertheless, the current repertoire of biomarkers for diagnostic and prognostic purposes in clinical settings is limited, underscoring the importance of research and development efforts to identify novel biomarkers that can effectively reduce cervical cancer in women.

The Egl-9 family (EGLNs) of hypoxia-inducible factors (HIFs) are tetramers of protein disulfide isomerase subunits and hydroxylase units. They are members of the oxygen-dependent ferrous and alpha-ketoglutaric acid dioxygenase superfamily [11]. The EGLN family, comprising EGLN1, EGLN2, and EGLN3, plays a pivotal role in cancer biology beyond its primary function to regulate HIF factors [12]. These enzymes function as oxygen sensors and control HIF-α subunit degradation under normoxic conditions [13]. However, their activity extends to regulate crucial cellular processes, including survival, metabolism, proliferation, and transcription, frequently *via* non-HIF targets and hydroxylase-independent mechanisms [14]. EGLN enzymes exhibit tumor-suppressive and tumor-promoting effects depending on the specific isoform and tumor type. For instance, EGLN1, the most extensively studied gene, modulates the tumor microenvironment (TME), activates cancer-associated fibroblasts, and impedes HIF-1α-related angiogenesis, promoting cancer metastasis [15, 16]. The diverse and occasionally conflicting functions of EGLNs across various cancers emphasize the need for additional research to clarify their specific mechanisms of action [12]. Recent progress in comprehending EGLN activity has generated interest in the potential targeting of these proteins for cancer therapy [17]. Nevertheless, EGLN inhibitors should be evaluated individually due to their intricate roles in cancer progression [18, 19]. In summary, EGLN prolyl hydroxylases are promising targets for cancer therapies. However, a comprehensive understanding of their roles in various cancers is crucial for developing effective therapeutic strategies.

This research aimed to analyze the mutation profiles of EGLNs in diverse human cancers. Afterwards, we investigated the expression levels of EGLN1, EGLN2, and EGLN3, along with their association with cancer prognosis. Additionally, we evaluated the connection between EGLN expression and immune subtypes, immunotherapy responses, TME scores, as well as cancer stemness in various human cancers. We investigated EGLN expression in multiple public single-cell datasets across pan-cancer cell types. Additionally, the EGLN score was developed using three EGLN genes and single-sample gene set enrichment analysis (ssGSEA) to evaluate collective functions of EGLNs in various cancers. Subsequently, expression level and prognostic significance of EGLN1 in cervical cancer were validated using public databases and clinical specimens. This study represents the first attempt to elucidate and provide an overview of the roles of EGLNs in pan-cancer. Besides, our study contributes to a deeper understanding of the molecular features of EGLNs and their potential applications as prognostic biomarkers. We also integrated various public databases and datasets to provide a multiomics-based analysis of EGLNs in pan-cancer, including validating the important role of EGLN1/2/3 as promising biomarkers in cervical cancer using clinical samples.

## 2 MATERIALS AND METHODS

### 2.1 Data Resources and Patients

The gene expression profiles, mutation data, stemness scores, and clinical information were retrieved from The Cancer Genome Atlas (TCGA) utilizing the UCSC Xena platform (https://xenabrowser.net/). Data pertaining to normal tissues, including para-cancerous samples, were sourced from the Genotype-Tissue Expression (GTEx) project. (https://gtexportal.org/home/index.html). Furthermore, the Therapeutically Applicable Research to Generate Effective Treatments (TARGET, https://www.cancer.gov/ccg/research/genome-sequencing/target) initiative and the Gene Expression Omnibus (GEO, https://www.ncbi.nlm.nih.gov/geo/) database were employed to corroborate the prognostic implications of EGLNs across various cancer types.

In this investigation, tumor samples and their corresponding normal tissue counterparts were prospectively and randomly collected from 10 individuals diagnosed with cervical squamous cell carcinoma who underwent surgical treatment at Huaihe Hospital of Henan University from 01/01/2020-12/30/2023. Participants have provided informed consent in written form. The study enrolled patients who fulfilled the following criteria: (1) a confirmed pathological diagnosis of cervical cancer, (2) the receipt of radical surgical resection, and (3) the availability of comprehensive clinicopathological and follow-up data. Patients were excluded if they had received preoperative chemotherapy or radiotherapy, had concurrent malignancies affecting other organs, or if their surgical resection was deemed non-curative with positive margins upon pathological examination. We have de-identified all patient details, and the age and gender of patients are indicated in Table S1. The study protocol was approved by the Ethics Committee of Huaihe Hospital of Henan University and was conducted in accordance with the ethical principles outlined in the Declaration of Helsinki. The reporting of this study conforms to REMARK guidelines [20].

### 2.2 Genomic Alteration Analysis of EGLNs in Cancers

The cBioPortal (http://www.cbioportal.org/), a tumor genome exploration platform, was applied to investigate oncogenic genomic alterations in EGLN genes across all cancer types [21]. Various mutations, such as amplification, missense, splicing, fusion, deep deletion, truncation, and in-frame mutations, were analyzed in the coding sequence among EGLN genes. Expression and methylation were analyzed using ggplot2 or data from the original source. Single nucleotide variation (SNV) was generated using maftools [22]. Copy number variation (CNV) plots were generated using GISTIC2.0 [23], followed by analysis using t-tests or analysis of variance. The integrated expression of the EGLN genes was calculated using gene set variation to compare data [24]. Additionally, the level 4 SNV dataset was processed by MuTect2 [25] from the GDC (https://portal.gdc.cancer.gov/), and mutation information from all patients was integrated. Structural domain of the EGLNs was obtained by the R package maftools.

### 2.3 Gene Expression and Survival Analysis of EGLNs in Cancers

Standardized cancer data for different cancers were downloaded from the UCSC platform. This data, known as TCGA TARGET GTEx (PANCAN, with 60,499 genes and 19,131 samples), served as our foundation. The TARGET database was used to identify diverse cancer categories and broaden our understanding. The Perl tool was used to investigate EGLN gene expression levels in these cancers. Then, a statistical test, “Wilcoxon test,” was applied to uncover how EGLN gene expression varied across different cancer types. However, for a more accurate analysis, cancer types with than five neighboring normal tissue samples were excluded (15 cancer types). Graphs, including box plots, heatmaps, and violin plots, were created using R packages “pheatmap” and “ggpubr” to visualize our findings. These results allowed us to identify patterns in EGLN gene expression. Additionally, another R package, “corrplot,” was applied to explore the relationships between the different EGLN genes. To acquire further insights, the cancer cell line encyclopedia database (https://sites.broadinstitute.org/ccle/) was used to explore the expression of EGLN family members in cancer cell lines. This allowed us to understand the behavior of these genes in a controlled laboratory setting.

The cBioPortal platform (http://www.cbioportal.org/) was employed to collect and organize survival data from patient samples in TARGET and TCGA databases and investigate the genetic regulation and impact on survival rates of the EGLN family genes. Subsequently, the relationship between EGLN gene expression and key survival metrics, including overall survival (OS), progression-free interval (PFS), disease-specific survival (DSS), and disease-free interval (DFS), was examined. Additionally, a Cox analysis was conducted to elucidate the correlations between EGLN expression and survival outcomes.

### 2.4 Tumor immune Score, Genomic Heterogeneity, and Tumor Stemness Analysis of EGLNs

The expression of the EGLN gene, relative to various immune scores, was meticulously analyzed using the R packages “ggplot2,” “limma,” and “reshape2.” To elucidate the relationship between EGLN gene expression and immune or stromal cells in cancer, the ESTIMATE algorithm [26] was utilized to compute immune, stromal, and cell scores. Spearman's correlation method was employed to investigate the association between EGLN family gene expression and RNA or DNA stemness scores (denoted as RNAss and DNAss, respectively). For visualization, these stemness relationships were depicted using plots generated with the R “corrplot” package. Subsequently, comprehensive nucleotide variation data (Level 4) for TCGA samples were retrieved from the GDC and processed using MuTect2. The tumor mutation burden (TMB) for each tumor was calculated using the “tmb” function from the R package “maftools” (version 2.8.05). These TMB data were then merged with the gene expression data, and a log2 (x + 0.001) transformation was applied to normalize each expression value. In a similar vein, microsatellite instability (MSI) scores for each tumor were derived based on previous studies [27]. These MSI scores were integrated with the gene expression data, and the same log2 (x + 0.001) transformation was applied to each expression value to maintain consistency throughout the analysis.

### 2.5 The Expression of EGLNs based on Multiple Single-cell Datasets

Single-cell sequencing was applied to study the genome (set of all genes), transcriptome (a gene that is actively being used), and epigenome (modifications that influence gene expression at the single-cell level) [28]. Gene expression data for various cancers were obtained at the single-cell level using the Tumor Immune Single Cell Hub (TISCH, http://tisch.comp-genomics.org) database [29]. A heatmap package was applied to visualize the complex EGLN family gene expression landscape across different cancers and single cells. A heatmap is a color-coded graphic that helps observe patterns and variations in the data. Hierarchical clustering was utilized to group similar patterns together, employing Euclidean distance as the metric for similarity and Ward's minimum variance method to optimize cluster distinctiveness. This approach makes it easier to identify patterns and trends in data, helping to understand that EGLN family gene expression is dysregulated in different cancers at the single-cell level.

### 2.6 Pathway and Enrichment Analysis of EGLNs

The significance of EGLNs was assessed in 10 key cancer-related biological processes, including cell division, cell death, tuberous sclerosis complex-mammalian target of rapamycin (TSC-mTOR) signaling, phosphoinositol-3 kinase (PI3K) /AKT signaling, receptor tyrosine kinase activity, RAt Sarcoma/Mitogen-activated protein kinase (RAS/MAPK) signaling, estrogen, androgen hormone receptor activity, DNA damage response, and the transformation of cells from one type to another during epithelial-mesenchymal transition (EMT). A pathway score for each process was calculated by summing the relative amounts of proteins that promote it and subtracting those that inhibit it. The pathway activity score (PAS) was determined as previously described [30], with a higher PAS for a gene in one group than another, indicating an activating role and a lower PAS suggests an inhibitory effect. GeneMANIA (http://genemania.org/) was used to analyze gene interactions, identify related genes [31], and predict that EGLNs may interact with other genes. This helped us understand their potential functional networks.

GEPIA2.0 was applied to search the top 20 genes most closely associated with EGLN1-3 across all human cancers and their normal counterparts and explore the relationships between EGLNs and other genes. Five datasets (EGLN1-3) were combined to gain insights into the biological processes, cellular locations, molecular functions, and metabolic pathways involved. These insights were visualized as bubble plots by R packages”ggplot2,” “tidyr,” and “clusterProfiler,” and making it easier to understand the complex interactions and functions of EGLNs and their related genes.

### 2.7 Construction of EGLN Score using ssGSEA

The EGLN score was computed using ssGSEA [24], focusing specifically on the EGLN gene set. This method helped measure gene expression levels for each cancer type. The EGLN scores were compared between tumor and normal tissue samples across multiple cancer types using TCGA database. Next, EGLN scores were integrated with survival data to investigate their potential as prognostic indicators in human cancers. Additionally, the relationship between EGLN score and different immune cells or immune checkpoints was analyzed to explore its broader implications in the TME.

### 2.8 Genetic Variation of EGLNs in Cervical Squamous Cell Carcinoma (CESC) using eQTL-GWAS Colocalization

Genome-wide association study (GWAS) data for cervical cancer were obtained from the GWAS Catalog (https://gwas.mrcieu.ac.uk/; Accession No. ieu-b-4876). GWAS data included 8,506,261 SNPs from 199,086 Europeans.

Bayesian total positioning analysis with the “coloc” package (https://github.com/chr1swallace/coloc) with its default settings was used to analyze the likelihood that two features share a common causal genetic variation. The ieugwasr_to_coloc function was used to extract colocalization data, while the coloc.abf function was used for genetic colocalization analysis of the two potentially linked phenotypes. This analysis aimed to determine whether they shared a common genetic cause within the genetic distance region of the relevant eqtl gene. A clear threshold was set for significant colocalization evidence, requiring PP.H4.abf to exceed 80%. The locus-compare function from the locus-compare package and the stack_assoc_plot function from the gassocplot2 package were used to visualize the results.

### 2.9 Validation and Immunohistochemistry of EGLN1 in CESC

In the context of a comprehensive pan-cancer prognosis analysis focusing on CESC, EGLN1 emerged as a notable gene with the highest hazard ratio (HR = 2.07, P = 1.1e-6). Consequently, EGLN1 and CESC were chosen for further validation. To assess EGLN1 expression in CESC patients, we downloaded data from TCGA CESC and the GTEx projects. EGLN1/2/3 expression levels were evaluated in unpaired CESC samples, complemented by corresponding samples from GTEx. Clinical data and RNA-sequencing expression profiles for CESC were retrieved from the TCGA database. Survival differences between groups were analyzed using the log-rank test. To evaluate the predictive accuracy of EGLN1/2/3 mRNA expression, we conducted time-dependent receiver operating characteristic (ROC) analysis. For Kaplan-Meier (KM) plots, P-values and HRs with 95% confidence intervals (CIs) were determined using univariate Cox proportional hazards regression and log-rank tests.

Tumor tissues for cervical cancer were prepared using conventional procedures involving paraffin embedding. Tissue sections were first embedded in paraffin, followed by baking. Subsequently, the slides underwent deparaffinization in xylene and were rehydrated using a series of graded alcohol solutions. Tissue sections were incubated with antibodies against EGLN1/3 (1:150 dilution; Proteintech) and EGLN2 (1:400 dilution; Abcam) at 4°C overnight. The slides were retrieved from the refrigerator and allowed to warm up to room temperature for 45 minutes. Following a thorough cleaning with PBS buffer, they were placed into the DAKO automatic immunohistochemistry instrument, specifically the Autostainer Link 48 model. In accordance with the “Autostainer Link 48 Usage Guide,” the appropriate procedures were chosen to execute the blocking, secondary antibody binding, and DAB color development steps. Subsequently, two pathologists, who were blinded to the sample identities, independently assessed the staining results based on both the staining intensity and the proportion of positive cells. The intensity scores were categorized as follows: 0 for negative, 1 for weakly positive, 2 for moderately positive, and 3 for strongly positive. The proportions of positive cells were scored as 0 for 0%, 1 for 1%-25%, 2 for 26%-50%, and 3 for more than 50%. The IHC score was then determined by adding together the intensity score and the proportion of positive cells.

### 2.10 Statistical Analyses

All data were statistically analyzed using tools specifically designed for databases and the R software program. In this study, the graphs and charts were created using database tools and R Studio (version 1.2.1335). All datasets were standardized by adjusting their means to zero to ensure consistency. The KM method, a widely used technique in survival analysis, was employed to evaluate the survival outcomes. Many testing approaches were applied to determine whether the group differences were statistically significant. A *P* < 0.05 was considered statistically significant for all our analyses, indicating a strong likelihood that the observed differences were not due to chance alone.

## 3 RESULTS

### 3.1 Genetic Alternation Profiles of EGLN family genes

**Fig. (1):**
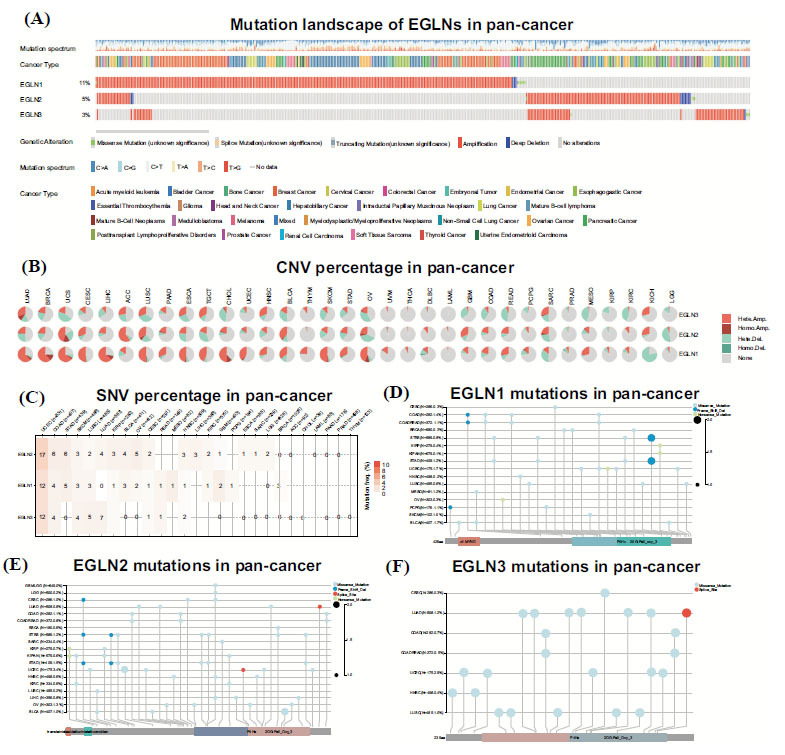
Genomic Profiles of EGLN Family Genes in Pan-cancer. (**A**) OncoPrint plot depicting the genetic alterations of EGLN genes across various human cancers. Each sample is represented by a column, while each EGLN gene is depicted by a row. The alterations are color-coded for clarity; (**B**) Pie chart illustrating the distribution of copy number variations (CNVs) across different cancers. Heterozygous amplification is represented by Hete Amp, heterozygous deletion by Hete Del, homozygous amplification by Homo Amp, and homozygous deletion by Homo Del. Samples without CNVs are indicated as None; (**C**) SNV landscape plot showing the distribution of single nucleotide variations (SNVs) in EGLN genes across all cancer types; D-F. Mutation landscape of EGLN1 (**D**), EGLN2 (**E**), and EGLN3 (**F**) genes across various cancer types.

Gene changes, including CNVs and SNVs, have been related to the development, growth, and treatment of tumors [32]. Fig. (**1A**) illustrates that EGLN genes vary across different cancer types. EGLN1 (13%), EGLN2 (5%), and EGLN3 (2%) exhibited significantly higher rates of genetic changes. The CNV analysis presents that EGKLN genes play different roles in various cancers. When we examined all cancers together, most CNVs in the EGLN gene group were heterozygous, meaning that one copy of the gene was changed, while a smaller number were homozygous, meaning that both copies were changed. LUAD, breast cancer (BRCA), and uterine carcinosarcoma (UCS) were the top three cancer types most strongly associated with CNV changes in EGLN genes (Fig. **1B**). Moreover, SNV analysis revealed that EGLN1 was the most commonly mutated EGLN gene across 32 human cancers, especially in UCEC, which was mutated in 17% of cases. When examining the mutations in EGLN1-3, we found that most were evenly spread across the parts of EGLN genes that code for proteins (Fig. **1C**). Among the 37 cancers, CESC, LUSC, and COAD affected all the EGLN genes. Additionally, 16 cancers exhibited changes in EGLN1 expression, which determines its structure (Figs. **1D**-**F**).

### 3.2 Pan-cancer Expression Profiles of EGLN Family Genes

**Fig. (2):**
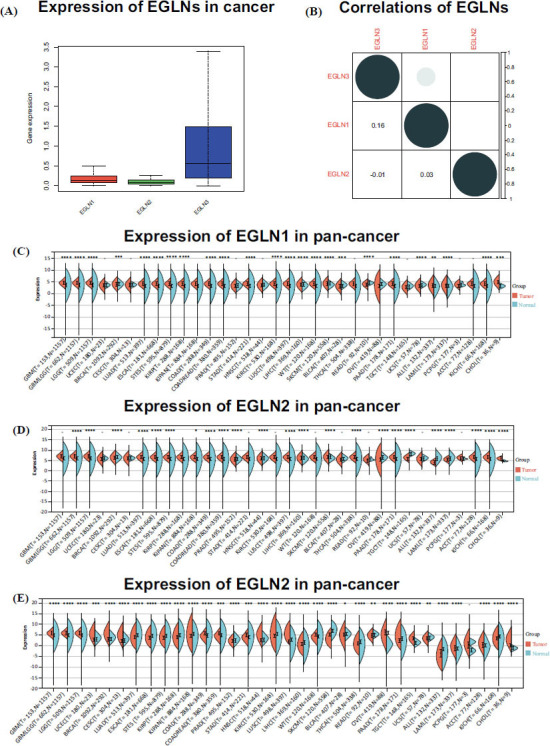
Expression Profiles of EGLN Family Genes Across Various Cancer Types. (**A**) Box plots depicting the relative expression levels of EGLN genes in a pan-cancer analysis; (**B**) Matrix graph displaying the Pearson's correlation coefficients of EGLN gene expression across different cancer types. Blue dots signify positive correlation, while red dots indicate negative correlation; C-E. Expression landscape of EGLN1 (**C**), EGLN2 (**D**), and EGLN3 (**E**) genes in various cancer types. *p < 0.05, **p < 0.01, ***p < 0.001, ****p < 0.0001.

Initially, we examined EGLN family members expressed in different cancer cells and discovered that all EGLNs were more active in these cells than usual (Figs. **S1A-C**). We thoroughly analyzed three EGLN family members in 33 human cancers using data from the TCGA database to gain a broader understanding of EGLN expression across various cancers. Our findings revealed that EGLN3 exhibited the highest expression levels, whereas EGLN2 exhibited the lowest across all cancers (Fig. **2A**). EGLN family genes tend to be overexpressed compared to their levels in healthy tissue surrounding the cancers. Moreover, we found that the expression levels of EGLN family members presented very weak or barely noticeable connections in different cancers, with a correlation index ranging from −0.01 to 0.16 (Fig. **2B**). These findings suggest that EGLN genes exhibit distinct expression patterns that vary within the EGLN family and among different cancers. Determining whether a specific EGLN gene acts as a cancer-promoting or cancer-suppressing factor is complex and depends on the cancer type. Accordingly, further investigation of the individual expression patterns of each EGLN gene is necessary.

Gene expression varies significantly among different subtypes of the same cancer [33]. We combined the TCGA, GTEx, and TARGET data to validate our findings. Our validation analysis confirmed that the expression levels of the three EGLN genes were significantly changed in various cancers (Figs. **2C**-**E**). Specifically, EGLN1 was overexpressed in 15 cancer types and downregulated in 9 types (Fig. **2C**). EGLN2 expression was higher in 9 cancer types and lower in 14 types (Fig. **2D**). Meanwhile, EGLN3 expression was higher in 21 cancer types and lower in 12 types (Fig. **2E**). These results reinforce our understanding of the complex and variable expression patterns of EGLN genes in cancer.

### 3.3 Patient Survival Correlated with the Expression of EGLN Family Genes

**Fig. (3):**
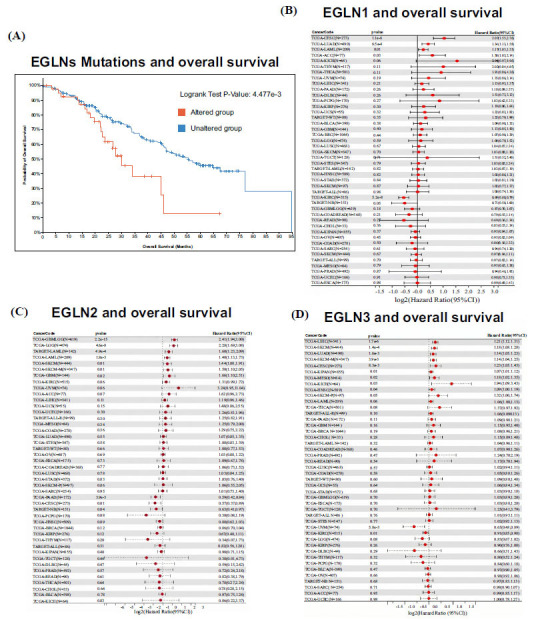
Correlation of EGLN genes expression with patient’s survival. A. Kaplan-Meier survival curves were generated to assess the association between EGLN gene alterations and overall survival. The results indicated that patients with altered EGLN genes had a poorer prognosis across various cancer types; B-D. Forest plots were created to illustrate the findings of the univariate Cox proportional hazards model, which evaluated the correlation between the expression levels of EGLN1 (B), EGLN2 (C), and EGLN3 (D) and overall survival in a diverse group of cancer patients.

A comprehensive study explored the changes in the expression patterns of EGLN genes that might impact the survival outcomes of patients with different cancer types. EGLN genes, which can vary greatly in their genetic makeup and activity levels, are associated with patient survival. Our research revealed that alterations in EGLN genes were significantly associated with worse OS and progression-free survival in various cancers (Fig. **3A**). We discovered that survival risk was closely associated with EGLN gene expression levels using a statistical model, the Cox model. Specifically, when we applied the Cox proportional hazards model, we observed a clear connection between EGLN gene expression and the likelihood of patient survival (Figs. **3B**-**D**). However, specific EGLN genes that act as risk factors for better or worse survival can differ depending on the cancer type. For instance, high EGLN1 expression increased the risk of poor survival in four cancer types (CESC, LUAD, LAML, and ACC) but reduced the risk of KIRC (Fig. **3B**). Similarly, high EGLN2 expression was beneficial for OS in seven cancer types, while low expression was associated with worse survival in CESC, NB, and PAAD (Fig. **3C**). However, high EGLN3 expression was linked to survival risk in several cancer types, including CESC, LUAD, and kidney cancer (Fig. **3D**). In summary, the patterns of EGLN gene expression emerged as strong indicators of patient survival, with these associations varying according to the specific cancer type being considered.

### 3.4 EGLN Family Genes were Associated with TME, Tumor Stemness, and Genome Heterogeneity

**Fig. (4):**
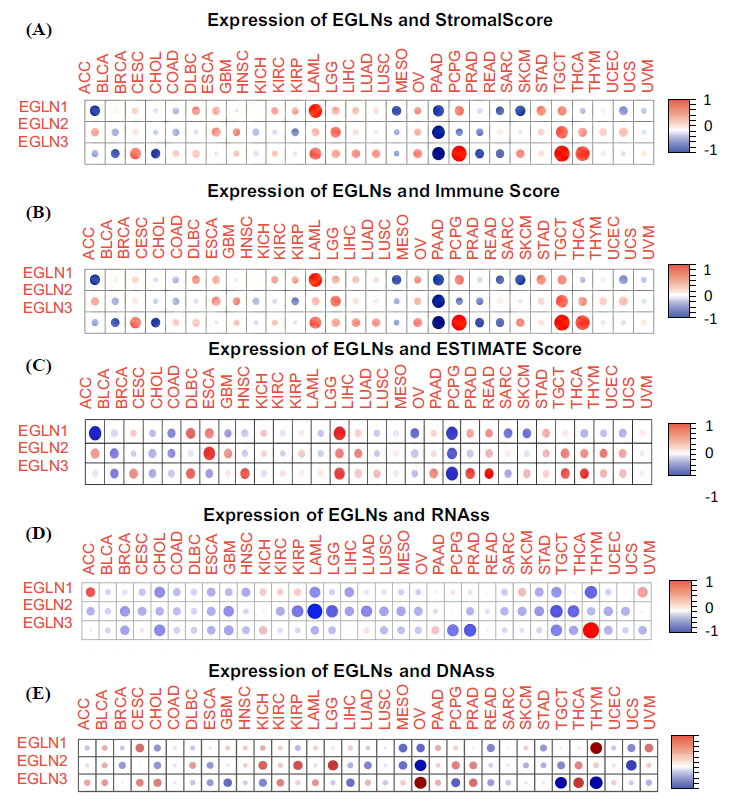
The correlation between EGLN genes expression and TME/ DNAss/RNAss in pan-cancer. A-C. Correlation matrices depicting the relationship between EGLN gene expression and various cancer-related scores: StromalScore (**A**), ImmuneScore (**B**), and ESTIMATEScore (**C**) across different cancer types. The cancer types and EGLN genes are displayed on the horizontal and vertical axes, respectively. Red signifies a positive correlation, whereas blue indicates a negative correlation; D-E. Correlation matrices showcasing the association between EGLN gene expression and cancer stemness scores: RNAss (**D**) and DNAss (**E**) in various cancer types. The cancer types and EGLN genes are represented on the horizontal and vertical axes, respectively. Red denotes a positive correlation, while blue signifies a negative correlation. RNAss stands for RNA stemness score, and DNAss stands for DNA stemness score.

Tumors have become complex environments, comprising supporting tissues (stromal), cancer cells, and immune cells that infiltrate them. Researchers have found that certain genes, EGLN1, EGLN2, and EGLN3, are linked to active surrounding tissues and immune cells in most cancer types (Figs. **4A**-**C**). Particularly, EGLN1 exhibited a consistently positive relationship with this activity across all cancers, while EGLN3 exhibited a consistently negative connection. These findings demonstrate that each EGLN gene affects the tumor environment differently.

Understanding the interaction of EGLN genes with the immune system is crucial because it may help to identify cancers that respond to immunotherapies targeting EGLN. Additionally, cancer stem cells drive aggressive behavior, metastasis, resistance to treatment, and cancer recurrence. In this study, we explored the relationship between EGLN expression and stem-like cancer cells, including both RNA and DNA markers [34]. We examined various cancers and found that EGLN genes were positively and negatively associated with stem-like markers. EGLN1, EGLN2, and EGLN3 were frequently negatively linked to one of these markers (RNAss) in 32 cancer types, although the relationship between EGLN1 and RNAss was weaker across all cancers (Fig. **4D**). Meanwhile, EGLN2 was positively associated with other markers (DNAss) in some cancers (Fig. **4E**). Although there are different ways to measure stem-like traits in cancer cells, our study highlighted that the EGLN gene is related to these traits in different tumor types to varying degrees. This underscores the complexity of cancer and the need for targeted approaches to cancer therapy.

**Fig. (5):**
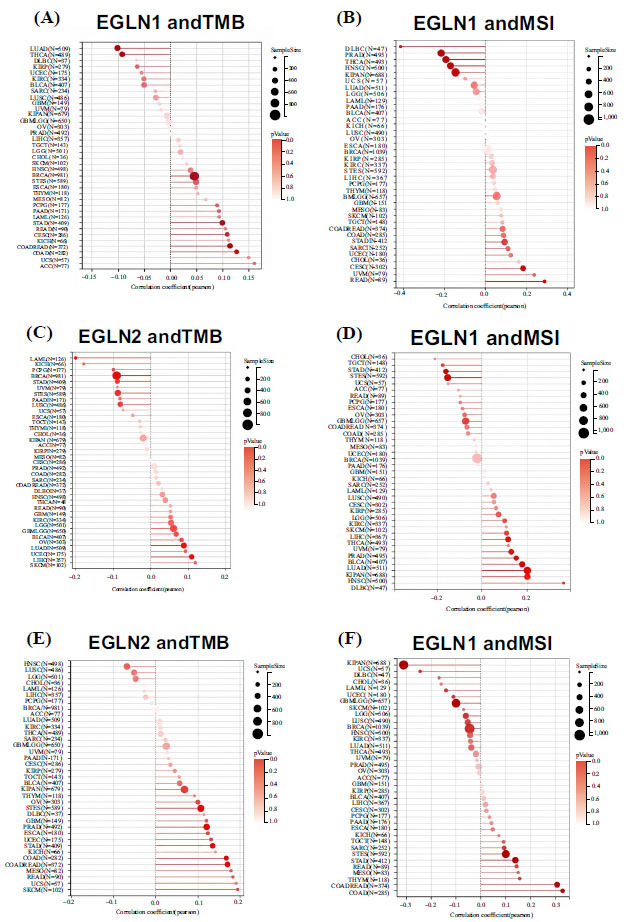
The correlation between EGLN genes expression and TMB and MSI in pan-cancer. (**A-B**) The correlation between EGLN1 expression and TMB and MSI in pan-cancer; (**C-D**) The correlation between EGLN2 expression and TMB and MSI in pan-cancer; (**E-F**). The correlation between EGLN3 expression and TMB and MSI in pan-cancer.

Regarding the association of EGLNs with TMB and MSI scores, we observed that EGLN1 was positively related with three tumors, COAD (R = 0.13, *P* = 0.03), colon adenocarcinoma/rectum adenocarcinoma (COADREAD) (R = 0.11, *P* = 0.02), and stomach adenocarcinoma (STAD) (R = 0.099, *P* = 0.045), and negatively associated with LUAD (R = −0.1, *P* = 0.02) and thyroid carcinoma (THCA) (R = −0.09, *P* = 0.04). Besides, high EGLN1 expression was positively related to high MSI scores in three tumors, while low MSI indicators in five tumor types (Figs. **5A**-**B**). Similarly, the association between EGLN2-3 and TMB or MSI scores was validated in pan-cancer (Figs. **5C**-**F**). Their results revealed that EGLNs may be potential biomarkers for predicting genomic heterogeneity.

### 3.5 The Expression of EGLNs Based on Multiple Single-cell Datasets

**Fig. (6):**
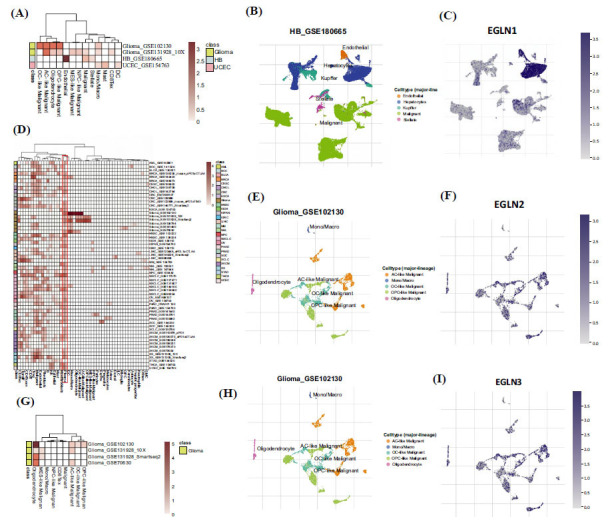
The EGLN genes expression in single cell level across pan-cancer. (**A**) The EGLN1 expression in single cell level across pan-cancer; (**B**) single cell expression level in HB based on GSE180665; (**C**) EGLN1 expression in single cell level of HB; (**D**) The EGLN2 expression in single cell level across pan-cancer; (**E**) single cell expression level in glioma based on GSE102130; **(F**) EGLN2 expression in single cell level of glioma; (**G**) The EGLN3 expression in single cell level across pan-cancer; (**H**) single cell expression level in glioma based on GSE102130; (**I**) EGLN3 expression in single cell level of glioma. The rows correspond to distinct datasets, with consistent labeling colors assigned to identical disease types. The columns denote various cell types. The color bar on the right serves as a reference for the correlation between data values and their corresponding colors, elucidating the magnitude of the data as indicated by the color gradients in the graph. A darker hue (red) signifies a higher data value, while white indicates cells that are either expressed as zero or not detected in the current dataset.

After excluding datasets with low EGLN expression across all cell types (defined as an average expression level of > 1), the expression patterns of EGLNs were analyzed to mitigate the risk of false-positive results. The data revealed that EGLN1 was predominantly expressed in malignant and endothelial cells, with a particularly high expression in hepatoblastoma. Figs. (**6A**-**C**) illustrate elevated EGLN1 expression in the endothelial cells of hepatoblastoma, suggesting that targeting these cells is a promising therapeutic strategy for treating hepatoblastoma. Furthermore, EGLN2 expression in monocytes/macrophages was consistently upregulated across various single-cell datasets for multiple human cancers (Fig. **6D**). Notably, elevated EGLN2 expression in monocytes/macrophages suggested a role in the pathogenesis of glioma cells (Figs. **6E**-**F**). In contrast, EGLN3 expression was predominantly regulated in gliomas (Fig. **6G**). EGLN3 expression in oligodendrocytes was significantly upregulated in gliomas (Figs. **6H**-**I**).

### 3.6 Expression Pattern of EGLN Family-related Genes

**Fig. (7):**
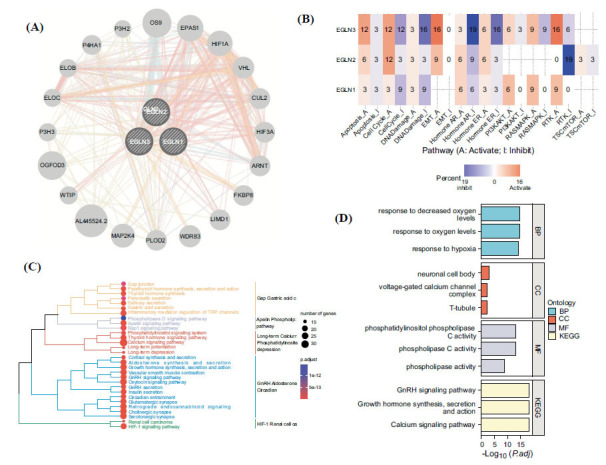
The cross-talk profile and enrichment analysis of EGLN and EGLN- associated genes. (**A**) The interaction networks of EGLN genes; (**B**) Heatmap of the percentage of the effect of EGLN genes on cancer pathway activity. Each pathway (activate or inhibit) was represented as a column and each EGLN gene was represented as a row; C-D. The KEGG (**C**) and GO (**D**) enrichment analysis of EGLN and EGLN-associated genes.

We performed an in-depth analysis of the interconnected network of the EGLN family to comprehensively understand the functional mechanisms of EGLNs and their associated proteins. This visualization identified that EGLN genes interact with many other genes to regulate cellular behavior and disease progression. These interactions occur through various mechanisms, including physical and genetic interactions, shared expression profiles, colocalization, common pathways, and protein characteristics (Fig. **7A**). Afterwards, we carried out a comprehensive examination of the interplay between EGLN gene activity and ten pathways frequently linked to cancer. Our results revealed a notable association between EGLN expression levels and the regulation of these cancer-relevant pathways, as illustrated in Fig. (**7B**). It is important to note that the exact influence exerted by EGLN genes on these pathways may vary according to the specific type of cancer and the unique attributes of each pathway involved. Our investigation revealed that elevated EGLN1 levels were significantly correlated with EMT activation in 16% of cancer cases, underscoring its critical role.

We analyzed the potential molecular mechanisms of the EGLN family using enrichment analysis. We identified the top 30 genes correlated with EGLN1, EGLN2, and EGLN3 expression by integrating expression data from 33 TCGA tumor datasets using GEPIA2.0. Then, we aggregated these EGLN-correlated genes (100 genes) to perform Kyoto encyclopedia of genes and genomes (KEGG) and gene ontology (GO) enrichment analyses. These findings revealed that most genes were implicated in cell cycle-related processes, including HIF-1α and gonadotrophin-releasing hormone (GnRH) -related signaling pathways (Fig. **7C**). Moreover, our study revealed a strong association between EGLN-correlated gene expression and responses to varying oxygen levels, including hypoxic and decreased oxygen conditions. EGLN-associated genes were significantly correlated with neuronal cell bodies at the cellular component level. KEGG pathway analysis indicated that EGLN-correlated genes were predominantly involved in the GnRH signaling pathway, growth hormone synthesis, secretion, and action, as well as the calcium signaling pathway (Fig. **7D**). These results suggest that EGLNs and their correlated genes may be critical in regulating responses to oxygen levels *via* essential components.

### 3.7 Construction of EGLN score based on ssGSEA

**Fig. (8):**
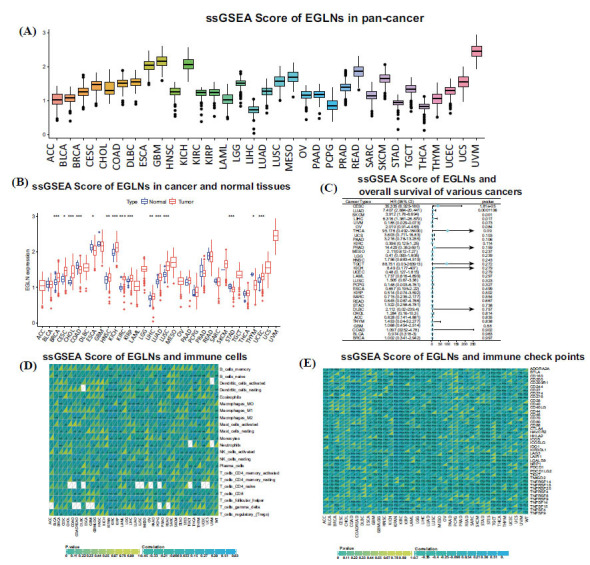
Construction of EGLN score using ssGSEA. (**A**) ssGSEA Score of EGLNs in pan-cancer; (**B**) ssGSEA Score of EGLNs in cancer and normal tissue; (**C**) association between ssGSEA Score of EGLNs and overall survival in various cancers; (**D**) relationship between ssGSEA Score of EGLNs and immune cells in pan-cancer; (**E**) relationship between ssGSEA Score of EGLNs and expression of immune checkpoints in pan-cancer.

Fig. (**8A**) depicts the varying levels of EGLN score expression among different cancer types, notably showing the highest score in uveal melanoma (UVM). When compared to normal tissues, it was found that the EGLN score was markedly increased in 11 cancer types and decreased in 4 types, as displayed in Fig. (**8B**). Additionally, a notably high EGLN score was found to be significantly associated with a poor prognosis in patients diagnosed with CESC (HR = 35, *P* = 1.81e-5), underscoring the prognostic significance of EGLN in CESC. The EGLN score was associated with various immune cells and checkpoints across different human cancer types (Figs. **8C**-**E**).

### 3.8 Genetic Variation and Prognostic Values of EGLNs in CESC

**Fig. (9):**
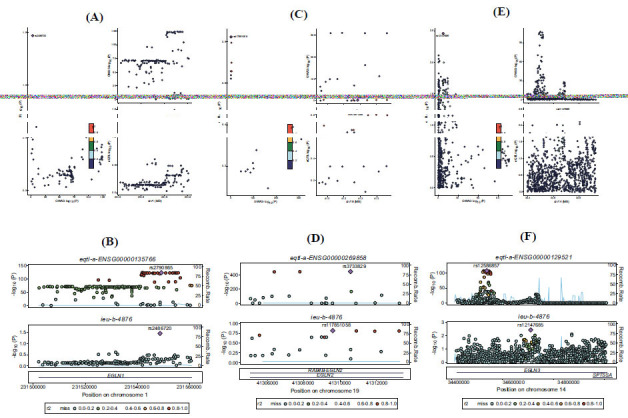
Colocalization analysis of eQTL-GWAS between EGLNs and cervical cancer. (**A**) Colocalization analysis of eQTL-GWAS between EGLN1 and cervical cancer using the locuscompare R package for visualization. The labeled SNP is the lead SNP (in this case for both studies), and other SNPs are colored according to their Linkage Disequilibrium (LD) with the lead SNP; (**B**) Colocalization analysis of eQTL-GWAS between EGLN1 and cervical cancer using the gassocplo R package for visualization. Based on GRCh37 (hg19) or GRCh38 (hg38) coordinates, the stacking regional association maps of eQTL and GWAS traits in genomic regions are plotted. The purple diamond represents the minimum P value SNP corresponding to eQTL and GWAS; (**C**) Colocalization analysis of eQTL-GWAS between EGLN2 and cervical cancer using the locuscompare R package for visualization; (**D**) Colocalization analysis of eQTL-GWAS between EGLN2 and cervical cancer using the gassocplo R package for visualization.; (**E**) Colocalization analysis of eQTL-GWAS between EGLN3 and cervical cancer using the locuscompare R package for visualization; (**F**) Colocalization analysis of eQTL-GWAS between EGLN3 and cervical cancer using the gassocplo R package for visualization.

The aforementioned analysis elucidated the role of EGLNs as potential biomarkers for CESC. Consequently, we further investigated the genetic variation in EGLNs in CESC using eQTL-GWAS colocalization. Figs. (**9A**, **B**, **E**, and **F**) indicate no shared genetic causal variation between EGLN1 and EGLN3 with CESC. However, a shared genetic causal variation between EGLN2 and CESC was identified, with an SNP.PP.H4 value of 0.999294116 (Figs. **9C**-**D**). Consequently, further validation of EGLNs in the context of CESC is warranted.

Patients with CESC were stratified into two groups based on their EGLN expression levels: those with high EGLN expression and those with low EGLN expression. As illustrated in Fig. (**10**), heightened EGLN expression was found to be linked with an elevated mortality risk in CESC patients. More specifically, individuals with high EGLN expression exhibited shorter survival durations compared to those with low EGLN1 expression. These results suggest that elevated EGLN expression could serve as a potential risk factor for CESC. To evaluate the prognostic value of EGLNs, time-dependent ROC curve analyses were performed. The area under the curve values for OS at one, three, and five years were determined to be 0.782, 0.763, and 0.827, respectively. Overall, EGLNs are strong candidate prognostic biomarkers in patients with CESC.

**Fig. (11):**
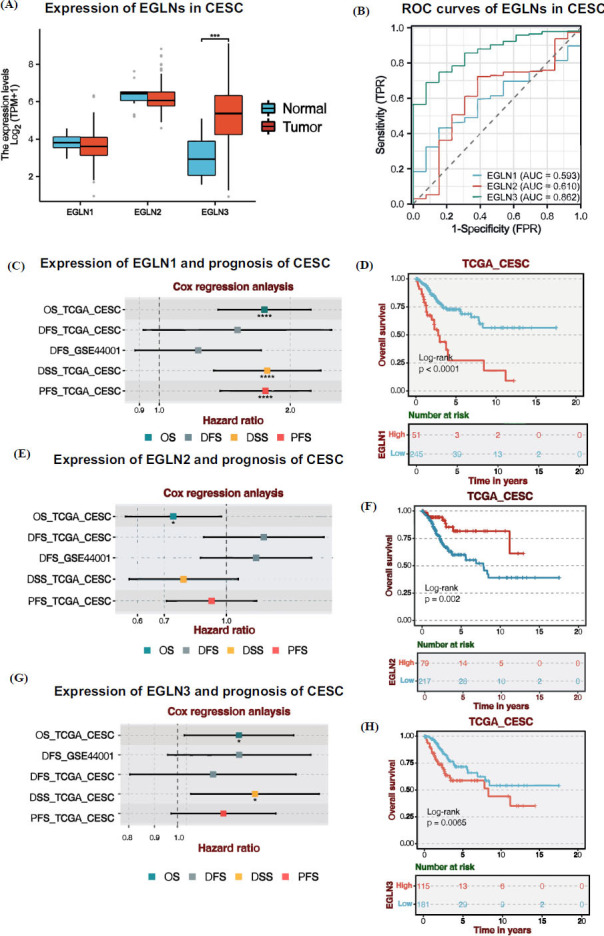
Further validation of the prognostic value of EGLNs in patients with CESC using integrated CESC cohorts. (**A**) Expression of EGLNs in CESC tissues compared with normal kidney tissues. Log2 (TPM + 1) was applied for log-scale; (**B**) ROC curves of EGLNs for CESC patients; (**C**) Correlation between EGLN1 and prognosis of patients with CESC using TCGA and GSE44001 cohorts; (**D**) Kaplan-Meier curves of EGLN1 and CESC patients’ overall survival; (**E**) Correlation between EGLN2 and prognosis of patients with CESC using TCGA and GSE44001 cohorts; (**F**) Kaplan-Meier curves of EGLN2 and CESC patients’ overall survival; (**G**) Correlation between EGLN3 and prognosis of patients with CESC using TCGA and GSE44001 cohorts; (**H**) Kaplan-Meier curves of EGLN3 and CESC patients’ overall survival. *p < 0.05, **p < 0.01, ***p < 0.001, ****p < 0.0001.

We observed that only EGLN3 was upregulated in CESC compared to normal tissues (Fig. **11A**). Additionally, the ROC value of EGLN3 in CESC was the highest among the three EGLNs (Fig. **11B**). We incorporated data from the GSE44001 dataset for external validation to further validate the prognostic significance of EGLNs in CESC. Figs. (**11C**-**D**) depict that elevated EGLN1 expression is a risk factor for patients with CESC. In contrast, high EGLN2 expression protects against OS in CESC. Moreover, increased EGLN3 expression was associated with poorer OS and DSS in patients with CESC (Figs. **11E**-**H**).

### 3.9 External Immunohistochemical Staining Validation of EGLNs in CESC

**Fig. (12):**
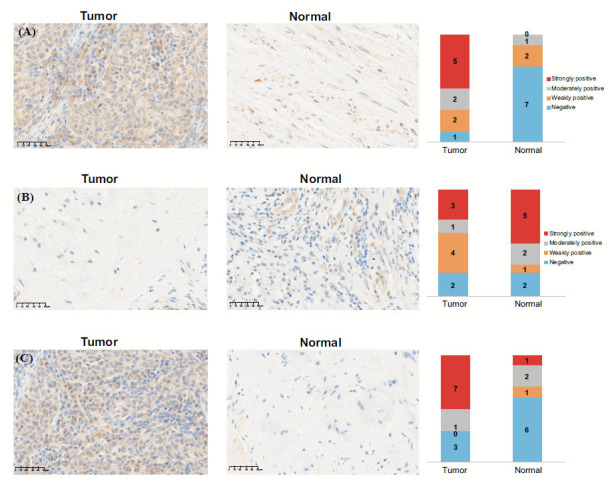
External immunohistochemical staining validation of EGLNs in CESC. (**A**) Immunohistochemical analysis of EGLN1 in 10 CESC tissue and normal tissue; (**B**) Immunohistochemical analysis of EGLN2 in 10 CESC tissue and normal tissue; (**C**) Immunohistochemical analysis of EGLN3 in 10 CESC tissue and normal tissue.

Immunohistochemical staining was conducted to verify the expression levels of EGLN1, EGLN2, and EGLN3. The findings confirmed that EGLN1 and EGLN3 were more abundant in CESC tissues compared to the adjacent normal tissues, while EGLN2 levels were lower in CESC tissues relative to the normal tissues. These results suggest that EGLNs have the potential to serve as biomarkers for CESC (Figs. **12A**-**C**).

## 4 DISCUSSION

This study provides a comprehensive pan-cancer analysis of EGLN family members (EGLN1, EGLN2, and EGLN3), focusing on their potential roles as biomarkers in cervical cancer. Our findings offer novel insights into the molecular mechanisms underlying EGLN expression and its prognostic significance in various cancers.

Hypoxia, a common cause of many malignant tumors, is closely associated with the progression of cervical cancer [35-37]. In response to hypoxic conditions, cancer cells undergo adaptive metabolic alterations, such as the conversion of glucose to lactic acid and the enhancement of glucose uptake through the upregulation of glucose transporters [17, 38]. Numerous studies have demonstrated that hypoxia can lead to metastasis and the development of resistance to radiotherapy, chemotherapy, and, potentially, molecular-targeted drugs and immunotherapy [38]. The transcription factor complexes HIF-1 and HIF-2 mediate the most typical molecular response to hypoxia, with the regulation of HIF-1α and HIF-2α primarily determining HIF activity [39]. HIF exerts regulatory control over prolyl hydroxylases PHD-1, PHD-2, and PHD-3, which in turn inhibit HIF activity. Among these, EGLN1 is the most crucial isoform for downregulating HIF under normoxic and mildly hypoxic conditions [40]. The EGLN1 expression and nuclear translocation make tumors more aggressive [41]. EGLN2, a prolyl hydroxylase that contributes to breast tumorigenesis, has recently been identified as a transcriptional co-activator through interaction with NRF1 to regulate mitochondrial function under normoxic and hypoxic conditions [42-44]. The EGLN3 expression was significantly decreased in various malignancies, including gastric cancer. However, the administration of EGLN3 hydroxylase pharmacologic inhibitors to mice bearing lung cancer impedes cancer growth by targeting the TME [45, 46]. However, the mechanisms underlying the expression and regulation of EGLN1/2/3 at the protein level in various cell types, its relationship with HIF-1 expression, and the association between EGLN1/2/3 expression and the clinicopathological characteristics of human cancers remain unclear.

Regarding how the EGLNs affect tumorigenesis or tumor inhibition, there was no study revealing the underlying mechanism. EGLNs, as a family of prolyl hydroxylase enzymes, exhibit remarkable specificity in influencing the progression of various cancer types, manifesting either tumor-suppressive or tumor-promoting roles that are contingent upon the EGLN isoform and the tumor type [47, 48]. This specificity is underscored by the intricate interplay between EGLNs and their targets, particularly the HIF (hypoxia-inducible factor) isoforms, which are pivotal in modulating the tumor microenvironment and cancer metastasis [18]. EGLN1, the most extensively studied isoform, has been shown to play a central role in modulating the tumor microenvironment by mediating the activation of cancer-associated fibroblasts and impairing HIF1α-related signaling, thereby promoting cancer metastasis [16]. This function is particularly evident in breast cancer, where EGLN1 activation leads to increased adenosine levels, indirectly upregulating c-MYC protein expression and contributing to tumorigenesis [49]. Conversely, EGLN2 and EGLN3 demonstrate a preference for modifying HIF2α, and their roles in cancer progression are context-dependent. For instance, EGLN2 overexpression in breast cancer directly represses HIF1α, decreasing cancer cell proliferation and formation [50]. The specificity of EGLNs in cancer progression is further highlighted by their interactions with other cellular factors. In lung cancer, EGLN1 and EGLN2 act as independent negative prognostic factors, with their higher expression correlating with poor patient outcomes [51]. Moreover, EGLNs demonstrate differential cellular localization in physiological and pathological conditions, which further contributes to their specificity in cancer progression [52]. For example, EGLN1 is predominantly expressed in the cytoplasm, while EGLN2 occupies the nuclear compartment. These localization patterns are subject to changes in many pathological conditions, influencing their interactions with target molecules and subsequent biological effects [53, 54]. In conclusion, EGLNs exhibit specificity in influencing cancer progression through their interactions with HIF isoforms and other cellular factors, as well as their differential cellular localization. Understanding the isoform- and context-specific roles of EGLNs in cancer will pave the way for the development of targeted therapies that harness their tumor-suppressive or tumor-promoting properties, ultimately improving patient outcomes.

Our analysis revealed that EGLN genes are more actively expressed in cancer cells than in normal tissues, with EGLN1 exhibiting the highest rates of genetic change among the EGLN family members. Notably, the expression patterns of EGLNs varied across different cancer types, suggesting that their roles in cancer progression are context-dependent. This underscores the importance of investigating EGLN expression on a cancer-specific basis. Meanwhile, our results demonstrate a significant correlation between EGLN gene expression and patient survival outcomes. Specifically, alterations in EGLN genes were associated with worse OS and progression-free survival in various cancers. However, the prognostic impact of individual EGLN genes varied, with EGLN1 and EGLN3 generally emerging as risk factors for poor survival, while EGLN2 exhibited a protective effect in certain cancers. These findings highlight the potential of EGLNs as prognostic biomarkers and the need to evaluate their prognostic value in different cancers.

The presence of tumor-infiltrating immune cells within the tumor microenvironment (TME) has been linked to tumor progression, invasiveness, and responses to therapy [55, 56]. The extent of immune cell infiltration, the functionality of T cells, and the tumor mutation burden all play crucial roles in determining the sensitivity of tumors to immunotherapy [57]. EGLN genes were associated with multiple aspects of the TME, including immune infiltration, tumor stemness, and genome heterogeneity. For instance, EGLN1 consistently displayed a positive relationship with immune activity across all cancers, while EGLN3 demonstrated a negative connection. This suggests that EGLNs play diverse roles in modulating TME, which may have implications for cancer immunotherapy. Additionally, the association between EGLN expression and cancer stemness markers suggests that EGLNs may contribute to cancer aggressiveness and treatment resistance. Single-cell sequencing analysis provided insight into the cellular specificity of EGLN expression. EGLN1 was predominantly expressed in malignant and endothelial cells, particularly in hepatoblastoma, suggesting that targeting EGLN1 in these cells could be a promising therapeutic strategy. EGLN2 was consistently upregulated in monocytes/macrophages in various cancers, indicating its potential role in cancer-related inflammation. In contrast, EGLN3 expression was predominantly observed in gliomas, suggesting a specific function in these tumors.

We identified a complex network of genes interacting with EGLNs using gene interaction analysis, implicating their involvement in various cellular processes and pathways. Notably, EGLN-related genes were enriched in cell cycle-related processes, including the HIF-1α signaling pathway and responses to varying oxygen levels. These findings suggest that EGLNs may be critical in regulating cellular metabolism and adaptation to hypoxic conditions, common features of solid tumors [58].

In addition, the EGLN score, constructed using ssGSEA, was significantly upregulated in several cancer types compared to normal tissues. The association between EGLN score and survival outcomes further underscores the potential of EGLNs as prognostic biomarkers. Regarding tumor selection validation, this study selected the CESC samples to validate due to the prominent prognostic efficiency of EGLNs in CESC. Besides, many studies have reported that hypoxia plays a crucial role in the tumor microenvironment of cervical squamous cell carcinoma [59-61]. Some hypoxia-related biomarkers have shown high efficiency in multiple studies [35, 62, 63]. Therefore, we hypothesized that EGLNs, as the crucial mediators of HIFs, can be the potential biomarkers in cervical squamous cell carcinoma. So, this study selected 10 paired cervical squamous cell carcinoma tumor tissues and adjacent normal cervical epithelial tissues to validate our findings. In CESC, high EGLN1 expression emerged as a risk factor for poor survival, while EGLN2 displayed a protective effect. These findings provide a strong rationale for future validation studies to establish the clinical application of EGLNs as prognostic biomarkers for CESC. Genetic analysis revealed a shared causal variation between EGLN2 and CESC, supporting the potential involvement of EGLN2 in cervical cancer development. Furthermore, validation studies using immunohistochemical staining confirmed the prognostic value of EGLNs in CESC, with high EGLN1 expression correlating with shorter survival time. These results highlight the importance of EGLNs in cervical cancer and warrant further investigation of the underlying mechanisms.

More importantly, the drug development of EGLNs for the treatment of cancer is crucial, including cervical cancer [64, 65]. The development of anticancer therapeutics targeting the EGLN family necessitates a meticulous and interdisciplinary approach [66]. Initially, researchers must undertake comprehensive preclinical investigations to elucidate the biological functions and mechanisms of EGLN family proteins in cancer progression [67, 68]. This involves characterizing their interactions with other molecular pathways and identifying specific mutations or altered expressions that contribute to tumorigenesis. Following the validation of these targets, medicinal chemistry efforts are employed to design and synthesize small molecules or biologics capable of selectively binding to and inhibiting EGLN family members [69]. These compounds are subjected to rigorous *in vitro* and *in vivo* evaluations to determine their efficacy, selectivity, and toxicity profiles [70]. Concurrently, pharmacokinetic and pharmacodynamic studies are conducted to optimize drug delivery and dosing strategies. Collaboration with clinical teams is imperative for translating these findings into human trials, wherein the safety and efficacy of EGLN-targeting drugs are further assessed [71, 72]. Besides, nanocarriers that deliver EGLN-targeting drugs also needs to be verified due to the potential of safety and stability [73]. Throughout this process, continuous refinement and iteration based on experimental data and clinical outcomes are critical to achieving success in anticancer therapies targeting the EGLN family.

In comparison to prior research on specific cancers, our study possesses several notable advantages. While previous studies have concentrated on developing and validating prognostic signatures for particular cancer types, our study has taken a broader approach by focusing on pan-cancer data like other published studies [74]. This has rendered the EGLN score more versatile and widely applicable. Second, we identified the EGLN expression patterns at the single-cell level, such as in monocytes/macrophages and endothelial cells, suggesting novel therapeutic targets, especially hepatoblastoma. Third, strong correlations were found between EGLN gene expression and patient survival outcomes, establishing EGLNs as potential prognostic biomarkers in cervical cancer and other cancer types. We incorporated external datasets for validation and strengthened the credibility and reliability of our findings.

However, this study has several limitations that should be addressed in future studies. First, the study relied primarily on bioinformatic analysis and lacked direct experimental validation of the roles and mechanisms of EGLN genes in cancer progression. Additional functional studies are required to fully elucidate the mechanisms by which EGLN genes modulate cancer cell behavior and treatment response. Second, although the pan-cancer analysis was comprehensive, the analysis specific to cervical cancer may have been limited by the size and characteristics of the available patient cohort. Further validation in larger, more diverse populations is needed to ensure the robustness and applicability of the findings. Third, multiple factors drive cancer progression, and EGLN genes are only one aspect of this complex landscape. A complete picture requires a comprehensive evaluation of other genetic and epigenetic alterations.

## CONCLUSION

Our integrated pan-cancer analysis of EGLN family genes elucidated their multifaceted and heterogeneous roles in cancer biology. EGLN1, EGLN2, and EGLN3 demonstrate tumor-suppressive and tumor-promoting activities contingent upon specific cancer types and contextual factors. The complex interactions among EGLN expression, TME, and immune status highlight the necessity for further investigation to delineate the underlying mechanisms. EGLNs have been identified as potential prognostic biomarkers for cervical cancer, and their overexpression is significantly associated with poor survival outcomes. Our findings contribute to a deeper understanding of the involvement of the EGLN family in cancer and underscore the importance of further research in this area.

## Figures and Tables

**Fig. (10):**
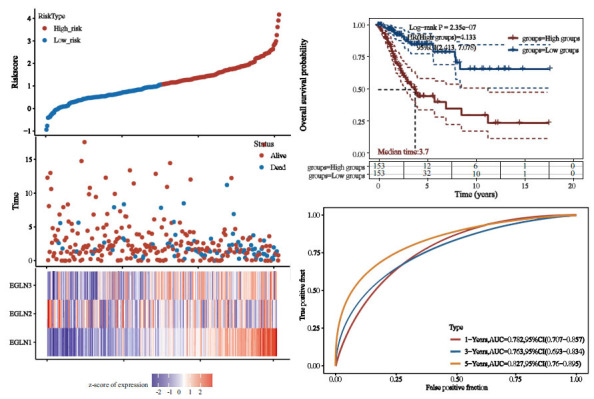
Further validation of the prognostic value of EGLNs in patients with CESC using TCGA cohort. The association between EGLN expression and patient survival is depicted as follows: The top panel displays a scatterplot where EGLN expression levels range from low to high. Different colors are used to distinguish between various expression groups. The middle panel presents a scatter diagram illustrating the distribution of survival time and survival status in relation to EGLN expression across different samples. The bottom panel features a heatmap representing the expression levels of EGLNs. Additionally, Kaplan-Meier curves are provided, showcasing the overall survival of patients with CESC based on EGLN expression levels using data from the TCGA CESC cohort. Furthermore, time-dependent ROC curves are displayed, demonstrating the relationship between EGLN expression and 1-, 2-, and 3-year OS rates for patients with CESC.

## Data Availability

The datasets presented in this study can be found in online repositories. The names of the repository/repositories and accession number(s) can be found in the article. Further inquiry can contact with corresponding authors.

## LIST OF ABBREVIATIONS

BRCA: Breast Cancer
CESC: Cervical Squamous Cell Carcinoma
CIs: Confidence Intervals
CNV: Copy Number Variation
DFS: Disease-free interval
DSS: Disease-specific Survival
EGLNs: Egl-9 Family
EMT: Epithelial-mesenchymal Transition
GEO: Gene Expression Omnibus
GnRH: Gonadotrophin-releasing Hormone
GO: Gene Ontology
GTEx: Genotype-Tissue Expression
GWAS: Genome-wide Association Study
HIFs: hypoxia-inducible Factors
KEGG: Kyoto Encyclopedia of Genes and Genomes
KM: Kaplan-Meier
LD: Linkage Disequilibrium
LUAD: Lung Adenocarcinoma
MSI: Microsatellite Instability
NB: Neuroblastoma
OS: Overall Survival
PAAD: Pancreatic Adenocarcinoma
PAS: Pathway activity Score
PFS: Progression-free Interval
RAS/MAPK: RAt Sarcoma/Mitogen-activated Protein Kinase
ROC: Receiver Operating Characteristic
SNV: Single Nucleotide Variation
ssGSEA: Single-sample Gene Set Enrichment Analysis
TARGET: The Therapeutically Applicable Research to Generate Effective Treatments
TCGA: The Cancer Genome Atlas
TISCH: Tumor Immune Single Cell Hub
TMB: The Tumor Mutation Burden
TME: Tumor Microenvironment
TSC-mTOR: Tuberous Sclerosis Complex-Mammalian Target of Rapamycin
UCS: Uterine Carcinosarcoma
UVM: Uveal Melanom
