# Supplementary material for: Comprehensive Pan-cancer Analysis and Experimental Verification of EGLN Family: Potential Biomarkers in Cervical Cancer
Source: Curr Cancer Drug Targets. 2025 Jun 3;26(2):176–97. doi: 10.2174/0115680096362252250527060004 (PMC13312406; doi:10.2174/0115680096362252250527060004)
Supplement: Supplementary file 1 [file CCDT-26-2-176_SD1.pdf]

Supplementary Material

Comprehensive Pan-cancer Analysis and Experimental Verification of EGLN Family: Potential Biomarkers in Cervical Cancer

Dongli Zhang[1,2,#], Ruifang Fu[2,#], Guixia Sun[2], Junfang Yan[2] and Xiaofeng Yang[1,*]

[1]Department of Obstetrics and Gynecology, The First Affiliated Hospital of Xi'an Jiaotong University, Xi'an, Shanxi 710061, P.R. China; [2]Department of Obstetrics and Gynecology, Huaihe Hospital of Henan University, Henan, Kaifeng 475000, P.R. China

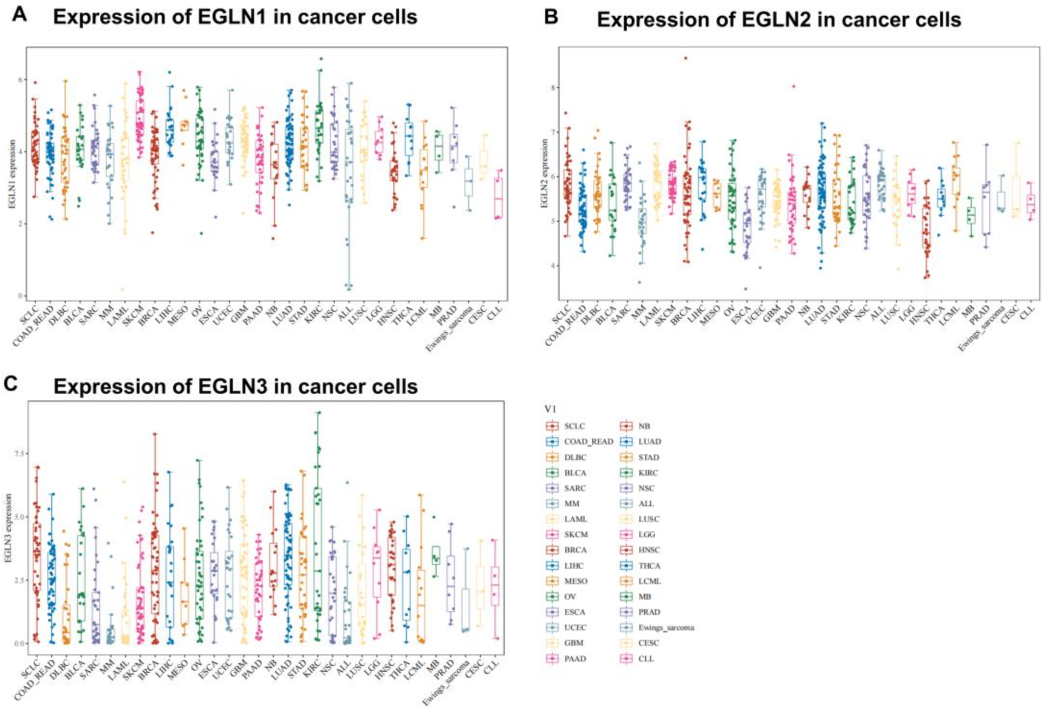

.

Fig. (S1). Expression Profiles of EGLN Family Genes Across Various Cancer Cell Lines. Expression landscape of EGLN1 (**A**), EGLN2 (**B**), and EGLN3 (**C**) genes in various cancer cell lines.
